# msCentipede: Modeling Heterogeneity across Genomic Sites and Replicates Improves Accuracy in the Inference of Transcription Factor Binding

**DOI:** 10.1371/journal.pone.0138030

**Published:** 2015-09-25

**Authors:** Anil Raj, Heejung Shim, Yoav Gilad, Jonathan K. Pritchard, Matthew Stephens

**Affiliations:** 1 Department of Genetics, Stanford University, Stanford, California, United States of America; 2 Department of Human Genetics, University of Chicago, Chicago, Illinois, United States of America; 3 Department of Biology, Stanford University, Stanford, California, United States of America; 4 Howard Hughes Medical Institute, Chevy Chase, Maryland, United States of America; 5 Department of Statistics, University of Chicago, Chicago, Illinois, United States of America; Albert Einsten College of Medicine, UNITED STATES

## Abstract

Understanding global gene regulation depends critically on accurate annotation of regulatory elements that are functional in a given cell type. CENTIPEDE, a powerful, probabilistic framework for identifying transcription factor binding sites from tissue-specific DNase I cleavage patterns and genomic sequence content, leverages the hypersensitivity of factor-bound chromatin and the information in the DNase I spatial cleavage profile characteristic of each DNA binding protein to accurately infer functional factor binding sites. However, the model for the spatial profile in this framework fails to account for the substantial variation in the DNase I cleavage profiles across different binding sites. Neither does it account for variation in the profiles at the same binding site across multiple replicate DNase I experiments, which are increasingly available. In this work, we introduce new methods, based on multi-scale models for inhomogeneous Poisson processes, to account for such variation in DNase I cleavage patterns both within and across binding sites. These models account for the spatial structure in the heterogeneity in DNase I cleavage patterns for each factor. Using DNase-seq measurements assayed in a lymphoblastoid cell line, we demonstrate the improved performance of this model for several transcription factors by comparing against the Chip-seq peaks for those factors. Finally, we explore the effects of DNase I sequence bias on inference of factor binding using a simple extension to our framework that allows for a more flexible background model. The proposed model can also be easily applied to paired-end ATAC-seq and DNase-seq data. msCentipede, a Python implementation of our algorithm, is available at http://rajanil.github.io/msCentipede.

## Introduction

A central challenge in modern genomics is the accurate identification of all the regulatory sequences that are active in a given cell type and a description of the mechanisms by which they regulate gene expression. One key mechanism is by recruiting transcription factors which bind to the DNA at characteristic nucleotide sequences. Chromatin immunoprecipitation followed by sequencing (ChIP-seq) provides a direct measurement of DNA sequences bound by transcription factors (either directly or through a co-factor); however, each ChIP-seq experiment provides information for only one transcription factor at a time. DNase-seq [1, 2] provides an indirect measurement of active regulatory sequences by exploiting the increased sensitivity of nucleosome-depleted chromatin to DNase I enzyme. While DNase-seq provides information on the active regulatory regions in the genome, identifying which transcription factors are bound to these regions and their organization requires statistical modelling of the spatial structure in DNase sensitivity in active regulatory regions [3–7].

Pique-Regi et al. [3] introduced a probabilistic framework to infer sequence motif instances that are bound by transcription factors, by combining sequence information with the information in DNase I cleavage patterns measured from DNase-seq assays. The model, CENTIPEDE, relies on two observations: (1) chromatin around motif instances bound by transcription factors typically has higher DNase I sensitivity than chromatin around unbound motif instances, and (2) each transcription factor has a characteristic DNase I cleavage profile around bound motif instances. Based on these observations, given a putative bound motif instance, CENTIPEDE models the number of reads mapped to each base pair along a window around the motif site as a mixture of two components (bound vs unbound), and infers the probability that each site is bound. Specifically, conditional on being bound (or unbound), CENTIPEDE models (1) the total number of DNase-seq reads using a negative binomial distribution, and (2) DNase-seq read counts along a window, conditional on the total number of reads, using a multinomial distribution, with independent sets of parameters for bound and unbound sites.

A limitation of the CENTIPEDE model is that it ignores variation in binding profiles across sites: it assumes that, given enough number of reads, the DNase I read count profiles would be the same at all bound sites, and that any variation in observed count profiles is due to multinomial sampling error from finite sequence coverage. However, in practice we have observed that read count profiles often have excess variation across factor-bound genomic locations and across replicate DNase-seq measurements compared with a multinomial model. Based on this, we hypothesized that improved modeling of this variation would improve predictions of transcription factor binding, particularly when multiple replicate DNase-seq datasets are available. Furthermore, when multiple replicate DNase-seq measurements are available for the same cell type, CENTIPEDE has often been applied after pooling replicates. If there is substantial heterogeneity between replicates, then pooling replicates tends to introduce more variation in the read count profiles, exacerbating the limitation of the multinomial model in this framework. The increasing availability of such replicate data [8] make improved performance in this setting particularly desirable.

**Fig 1:**
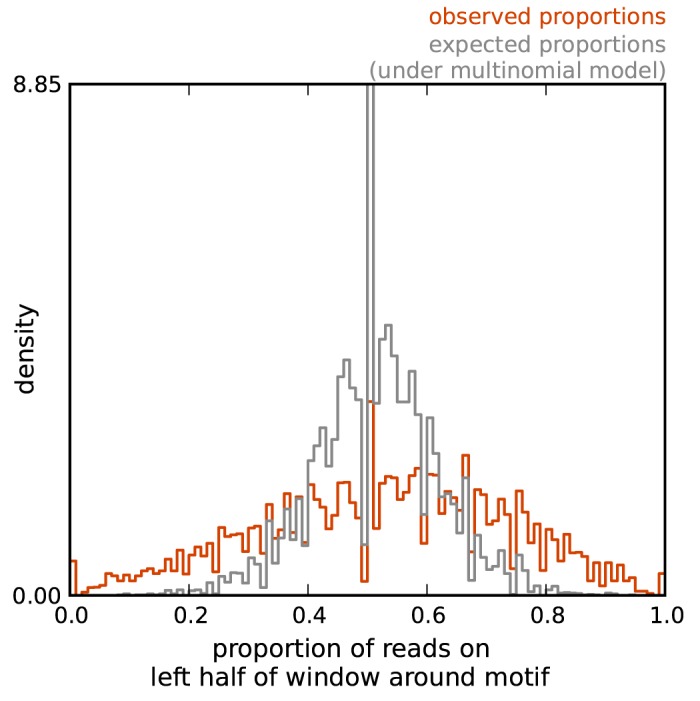
Illustration that DNase I cleavage profiles exhibit excess variation compared with a multinomial model. For a set of 1000 SP1 motif instances with high ChIP-seq signal, we computed, for a 100bp window around each motif instance, the ratio of number of DNase I cuts mapped to the left half of the window to the number of DNase I cuts mapped to the entire window. The histogram of these ‘observed ratios’ is shown in orange. Under a multinomial model the number of reads mapping to each half of the window should have a binomial distribution, and we used this fact to simulate ‘expected ratios’ (gray line); see S1 Methods for more details. The observed ratios are clearly overdispersed compared with the expectation under a multinomial model.

Fig 1 illustrates the excess variation in read count profiles noted above. The figure compares the distribution of the observed proportion of reads mapping to each half of a genomic window around each motif instance with its expectations under a multinomial sampling model (see S1 Methods for details). The distribution of observed (‘true’) proportions clearly exhibits a higher variance than expected under the multinomial model, demonstrating that multinomial sampling variation is insufficient to model the variation in read profiles across factor-bound genomic sites. Analagous plots at finer scales (smaller windows) show similar evidence for overdispersion (not shown).

Motivated by these observations, we have developed methods to better model heterogeneity in the read profiles across genomic locations and across replicate measurements of chromatin accessibility. Our methods are based on extending recent work using multi-scale methods for analyses of high-throughput sequencing data [9, 10]. A key feature of these multi-scale methods is that they allow for spatial structure in the heterogeneity across sites, with different amounts of variation at each spatial scale, and automatic identification of relevant scales during inference.

In addition to modelling heterogeneity among sites, the multi-scale methods provide a simple way to model the background cleavage model for DNase I. We have also implemented a flexible background model, and explored the improvement in performance when DNase-seq data from naked DNA are available to estimate its parameters.

## Methods and Data

Suppose we have *S* replicate DNase-seq measurements for a particular cell type or experimental condition. Consider a genomic window (site) of length *L* centered around each of *N* putative binding motifs, with *L* assumed to be a power of 2 (*L* = 2^*J*^). Let *X* ^*n*^ = (*X* ^*n*,1^, …, *X* ^*n*,*S*^), where $X^{n,s}={\left(X_{l}^{n,s}\right)}_{l=1}^{L}$ is the sequence of read counts in the *n* ^th^ site for the *s* ^th^ replicate and $X_{l}^{n,s}$ is read count at *l* ^th^ base pair in the site. Let *Z* ^*n*^ denote a binary indicator for whether the *n* ^th^ site is bound (*Z* ^*n*^ = 1). Following the model in CENTIPEDE [3], a mixture model at the *n* ^th^ site can be written as

$$\begin{array}{c} P\left(X^{n}\right)=P\left(X^{n}|Z^{n}=1\right)P\left(Z^{n}=1\right)+P\left(X^{n}|Z^{n}=0\right)P\left(Z^{n}=0\right), \end{array}$$ (1)

where

$$\begin{array}{c} P\left(X^{n}|Z^{n}=z\right)=\prod_{s=1}^{S}P\left(X^{n,s}|Z^{n}=z\right)\;\text{for}\;z=0,1 \end{array}$$ (2)

and the mixing proportion P(*Z* ^*n*^ = 1) = ζ_*n*_ is modeled as a logistic function of genomic information (e.g. motif position weight matrix score and motif sequence conservation score). Note that Eq (2) treats the *S* replicates for each site as independent given the bound/unbound status *Z* ^*n*^. In the following sections, we first detail our model for one replicate (here we drop the superscript ^*s*^) and then describe its extension to multiple replicates.

### msCentipede model at bound motifs

We modeled the profile of read counts at the *n* ^th^ site *X* ^*n*^ conditional on *Z* ^*n*^ = 1 using a Poisson model: $X_{l}^{n}∼Pois(\mu_{l}^{n})$ for *l* = 1, …, *L*. We allowed the mean read profile $\mu^{n}=(\mu_{1}^{n},\ldots ,\mu_{L}^{n})$ to vary across sites by using a hierarchical version of the multi-scale model for inhomogeneous Poisson processes introduced by Kolaczyk [11], and Timmermann and Nowak [12].

To introduce the ideas behind the multi-scale model, consider a single site with parameter vector *μ* = (*μ*_1_, …, *μ*_*L*_) (so drop the superscript ^*n*^ for simplicity). The key idea behind multi-scale Poisson models is to reparameterize this model in terms of parameters that capture spatial variation in *μ* at multiple scales, as follows. Let ${\left[\mu_{+}\right]}_{a}^{b}$ denote the sum $\sum_{j=a}^{b}\mu_{j}$. At the “zeroth” scale, define a single intensity parameter *λ*_0_ that captures the total intensity in the region

$$\begin{array}{c} \lambda_{0}:={\left[\mu_{+}\right]}_{1}^{L} \end{array}$$ (3)

At the first scale define a single parameter that captures the relative intensity in the first half of the region vs the entire region:

$$\begin{array}{c} p_{11}=\frac{{\left[\mu_{+}\right]}_{1}^{L/2}}{{\left[\mu_{+}\right]}_{1}^{L}}. \end{array}$$ (4)

At the second scale, define two parameters: one that captures the relative intensity in the first quarter of the region vs the first half; and one that captures the relative intensity in the third quarter vs the second half.

$$\begin{array}{c} p_{21}=\frac{{\left[\mu_{+}\right]}_{1}^{L/4}}{{\left[\mu_{+}\right]}_{1}^{L/2}};\;p_{22}=\frac{{\left[\mu_{+}\right]}_{L/2+1}^{3L/4}}{{\left[\mu_{+}\right]}_{L/2+1}^{L}}. \end{array}$$ (5)

At the third scale there are four parameters *p*_31_, …, *p*_34_ that similarly capture the relative intensity of an eighth of the region vs each quarter. This continues up to the *J*th scale (where recall *J* = log_2_(*L*)), in which there are *L*/2 = 2^*J*−1^ parameters of the form

$$\begin{array}{c} p_{J1}=\mu_{1}/\left(\mu_{1}+\mu_{2}\right);\;p_{J2}=\mu_{3}/\left(\mu_{3}+\mu_{4}\right);\;\cdots \end{array}$$ (6)

Combining across scales 0 to *J* this defines a total of *L* parameters, *p* = (*λ*_0_, *p*_11_, *p*_21_, *p*_22_, …, *p*_*J*(*L*/2)_), which are a one-to-one function of *μ*. That is, this defines a reparameterization of the model from *μ* = (*μ*_1_, …, *μ*_*L*_) to *p* = (*λ*_0_, *p*_11_, *p*_21_, *p*_22_, …, *p*_*J*(*L*/2)_).

This reparameterization has two key features: i) the likelihood P(*X*∣*p*) factorizes into a product form over the *L* elements of *p* (just as the likelihood P(*X*∣*μ*) factorizes into a product over the *L* elements of *μ*). Indeed, from elementary properties of the Poisson distribution, this factorization includes a Poisson likelihood for *λ*_0_ and a Binomial likelihood for each of the other parameters in *p*; see S1 Methods and [11] for details. ii) spatially-structured perturbations to the vector *μ* are captured by large perturbations in just a few elements of *p*. (By a spatially-structured perturbation, we mean a modification *μ*_*i*_ → *μ*_*i*_+*δ*_*i*_ such that *δ*_*i*_ tends to be similar to *δ*_*j*_ when ∣*i* − *j*∣ is small.) This property is related to the similar key property of wavelets [13], which are perhaps the best known multi-scale methods: spatially smooth signals tend to be concentrated into a small number of wavelet coefficients.

As a consequence of ii) we modeled spatially-smooth heterogeneity in *μ* ^1^, …, *μ* ^*N*^ across *N* putative binding sites using a simple hierarchical model for *p* ^1^, …, *p* ^*N*^ (where we have reintroduced superscript ^*n*^ to index sites). Specifically, we introduced parameters $\bar{p}=({\bar{\lambda }}_{0},{\bar{p}}_{11},{\bar{p}}_{21},{\bar{p}}_{22},\ldots ,{\bar{p}}_{J(L/2)})$ to represent the mean cleavage pattern across sites, and then assumed that site specific parameters *p* ^1^, …, *p* ^*N*^ are independent and identically distributed given $\bar{p}$, with

$$\begin{array}{c} \lambda_{0}^{n}|\bar{p},Z^{n}=1∼\text{gamma}\left(\alpha ,\alpha /{\bar{\lambda }}_{0}\right) \end{array}$$ (7)

$$\begin{array}{c} p_{jk}^{n}|\bar{p},Z^{n}=1∼\text{beta}\left({\bar{p}}_{jk}\tau_{j},\left(1-{\bar{p}}_{jk}\right)\tau_{j}\right) \end{array}$$ (8)

for *k* = 1, …, 2^*j*−1^ and *j* = 1, …, *J*, where *α* and *τ*_*j*_ are hyperparameters (estimated from the data) that control variability in the parameters at different scales. To ensure that the beta distributions in Eq (8) are unimodal, we constrain the hyperparameters $\left(\bar{p},\tau \right)$ such that ${\bar{p}}_{jk}\tau_{j}\geq 1$ and $(1-{\bar{p}}_{jk})\tau_{j}\geq 1$ for all *k* = 1, …, 2^*j*−1^ and *j* = 1, …, *J*.

### msCentipede model at unbound motifs

We modeled the read count profile at the *n* ^th^ site *X* ^*n*^ conditional on *Z* ^*n*^ = 0 using the same Poisson model, but with different distributions for the parameters:

$$\begin{array}{c} \lambda_{0}^{n}|Z^{n}=0∼\text{gamma}\left(\alpha^{o},\alpha^{o}/{\bar{\lambda }}_{0}^{o}\right) \end{array}$$ (9)

$$\begin{array}{c} p_{jk}^{n}|Z^{n}=0∼\delta_{0.5} \end{array}$$ (10)

where *δ*_0.5_ denotes the distribution with point mass on 0.5. Note that this means that $p_{jk}^{n}=0.5$, which is equivalent to assuming that the Poisson rates *μ* = (*μ*_1_, …, *μ*_*L*_) are all equal, resulting in uniformly distributed reads over the entire site. That is, it corresponds to the commonly-used assumption that there is no spatial structure in the read count profile when the transcription factor is not bound to its motif. Later, we propose a more flexible model for unbound sites.

### CENTIPEDE is a special case of msCentipede

The above msCentipede model (Eqs (9) and (10)) for unbound sites is exactly the same as the CENTIPEDE model for unbound sites. (The assumption of a gamma distribution for the Poisson rate parameter $\lambda_{0}^{n}$ in Eq (9) implies a negative binomial distribution for the total read-counts, which is exactly the model assumed by CENTIPEDE.) Furthermore, the msCentipede model for bound sites, Eq (8), becomes equivalent to the original CENTIPEDE model for bound sites in the special case *τ*_*j*_ → ∞, which corresponds to no heterogeneity in the shape of the cleavage pattern across bound sites. That is, msCentipede is an extension of CENTIPEDE to allow for heterogeneity in the shape of the cleavage pattern across bound sites.

### msCentipede for multiple replicates

When multiple replicates are available, msCentipede treats the replicates as independent (see Eq (2)). We assume the site and replicate specific parameters $p^{n,s}=(\lambda_{0}^{n,s},p_{11}^{n,s},p_{21}^{n,s},p_{22}^{n,s},\ldots ,p_{J(L/2)}^{n,s})$ for *n* = 1, …, *N* and *s* = 1, …, *S* (where we have reintroduced superscript ^*s*^ to index replicates) are independent and distributed as follows. Conditional on *Z* ^*n*^ = 1,

$$\begin{array}{c} \lambda_{0}^{n,s}|Z^{n}=1∼\text{gamma}(\alpha^{s},\alpha^{s}/{\bar{\lambda }}_{0}^{s}), \end{array}$$ (11)

where replicate-specific hyper parameters, *α* ^*s*^ and ${\bar{\lambda }}_{0}^{s}$, capture replicate-specific mean (${\bar{\lambda }}_{0}^{s}$) and variance ($\frac{{\bar{\lambda }}_{0}^{s2}}{\alpha^{s}}$). At the remaining scales,

$$\begin{array}{c} p_{jk}^{n,s}|Z^{n}=1∼\text{beta}\left({\bar{p}}_{jk}\tau_{j},\left(1-{\bar{p}}_{jk}\right)\tau_{j}\right), \end{array}$$ (12)

where hyper parameter ${\bar{p}}_{jk}$ represents the mean cleavage pattern across replicates and sites, and hyper parameter *τ*_*j*_ controls variability in the parameters at different scales.

This approach simplifies the problem by treating variation across replicates within a single site in effectively the same way as variation across sites within a single replicate. In principle this treatment could be improved—for example, by introducing a “random effect” at each site to represent site-specific variation that is shared across replicates. However, this would inevitably complicate the inference procedure, and we do not pursue it here.

The background model (*Z* ^*n*^ = 0) can be constructed in a similar way:

$$\begin{array}{c} \lambda_{0}^{n,s}|Z^{n}=0∼\text{gamma}(\alpha^{o,s},\alpha^{o,s}/{\bar{\lambda }}_{0}^{o,s}), \end{array}$$ (13)

$$\begin{array}{c} p_{jk}^{n,s}|Z^{n}=0∼\delta_{0.5}, \end{array}$$ (14)

where *δ*_0.5_ denotes the distribution with point mass on 0.5.

To account for the difference in the total number of sequence reads generated for each replicate, we allow for replicate-specific hyper parameters at the zeroth scale (see Eqs (11) and (13)). See S1 Methods for the computation of the likelihood.

### Flexible model for background DNase I cleavage rate

A number of studies have highlighted a strong sequence preference for DNase I cleavage [2, 14–16]. This sequence preference would cause the distribution of reads at unbound motif instances to be i) systematically non-uniform near the shared core motif; and ii) varying among motif instances away from the shared core motif (due to differences in the surrounding sequence). To account for these factors we consider a more flexible model for unbound sites. Specifically, we modify Eqs (10) and (14) as follows:

$$\begin{array}{c} p_{jk}^{n}|{\bar{p}}^{o},\tau^{o},Z^{n}=0∼\text{beta}\left({\bar{p}}_{jk}^{o}\tau_{j}^{o},\left(1-{\bar{p}}_{jk}^{o}\right)\tau_{j}^{o}\right), \end{array}$$ (15)

where the background parameters ${\bar{p}}_{jk}^{o}$ and *τ* ^*o*^ control the mean profile and the variance about this mean respectively. We estimated these background parameters using DNase-seq reads from naked DNA around the same set of motif instances, and refer to the method using this more flexible background model as msCentipede-flexbg. (In principle it is also possible to estimate these parameters using the DNase-seq data from chromatin, as part of the clustering of motif instances into bound and unbound motifs, but when we tried this we found msCentipede performed worse in practice than the uniform model (Eqs (10) and (14)), presumably because of the cost associated with attempting to estimate the many additional parameters of this more flexible model; see Discussion).

### Parameter estimation and inference

We estimated model parameters {*ζ*, *α* ^*s*^, ${\bar{\lambda }}_{0}^{s}$, *α* ^*o*,*s*^, ${\bar{\lambda }}_{0}^{o,s}$, *τ*_*j*_, ${\bar{p}}_{jk}$} by maximizing the likelihood across all putative binding sites using a variational optimization algorithm, accelerated using the SQUAREM method [17]. The variational optimization scheme is detailed in S1 Methods, and is equivalent to the expectation-maximization algorithm [18]. When DNase-seq data assayed in naked DNA were available, the background parameters ${\bar{p}}_{jk}^{o}$ and $\tau_{j}^{o}$ were first estimated using naked DNA assays; keeping these fixed, we, then, learned the remaining model parameters.

Inference on binding sites can be performed by computing the posterior odds for each site:

$$\begin{array}{c} \frac{P(n^{\text{th}}\;\text{site}\;\text{is}\;\text{bound}|X^{n})}{P(n^{\text{th}}\;\text{site}\;\text{is}\;\text{unbound}|X^{n})}=\frac{P(Z^{n}=1|X^{n})}{1-P(Z^{n}=1|X^{n})}. \end{array}$$ (16)

Detailed computation of P(*Z* ^*n*^ = 1∣*X* ^*n*^) is given in S1 Methods.

### Description of data and validation metrics

We executed msCentipede and CENTIPEDE using DNase-seq and ATAC-seq measurements assayed in the GM12878 lymphoblastoid cell line as data. Two replicate measurements using the UW DNase-seq protocol [2] and four replicate ATAC-seq measurements [19] were available for this cell line. The DNase-seq data were single-end reads that can be converted to counts of DNase I nicks for each base pair in a straightforward manner. The ATAC-seq data were paired-end reads; however, we ignored the information in the length of DNA fragments and used the counts of transpositions for each base pair as data.

We compared the algorithms on a set of 40 transcription factors with ChIP-seq data assayed by ENCODE in the same cell line [20], and for which PWM models were computed using data from high-throughput SELEX experiments [21, 22]. For each transcription factor, we identified a genomewide set of high-quality putative binding sites (PBS) using human genome reference GrCh37; for each PBS, the likelihood ratio for the PWM model vs a background model exceeded 1000. Using a 64 base-pair window around each PBS, we filtered out those sites that had fewer than 80% of bases in their window to be uniquely mappable. For each of the remaining sites, we computed the posterior probability that the transcription factor is bound, using CENTIPEDE and msCentipede. We used DNase-seq read count data from naked DNA derived from the IMR90 cell line [15] to fit the background model parameters in msCentipede-flexbg.

In addition, we compared the performance of msCentipede against that of PIQ [7] using DNase-seq data. Starting with a set of candidate binding sites, PIQ learns the background DNase I cleavage rate using a Gaussian process model. Then, PIQ estimates TF binding based on DNase I cleavage profiles and total DNase I cleavage rates that are specific to each TF, using the expectation propagation algorithm. We used the “score” metric output by PIQ as a measure of confidence of whether a motif instance is bound. When multiple replicate measurements are available, we executed PIQ by providing data from the replicates as separate input files.

We evaluated the accuracy of each of the three algorithms using Area under the Receiver Operating Curve (AuROC). To compute the AuROC, we selected a gold standard set of ‘bound motif instances’ and ‘unbound motif instances’; bound motif instances were PBS that lied within a ChIP-seq peak identified by a peak caller and ‘unbound motif instances’ were PBS that lied outside ChIP-seq peaks and had fewer ChIP reads than reads from a control IP experiment mapping to a 400 base pair window around the PBS, after controlling for total read depth. For each transcription factor, we executed two peak callers, MACS [23] and GEM [24], each with a 1% FDR cutoff, to generate two gold standard sets of bound and unbound motif instances. In this paper, we illustrate the accuracy of the algorithms evaluated against gold standards generated using GEM when using DNase-seq measurements as data. The accuracy of all three algorithms decreased by a modest amount when using the gold standards generated by MACS (see S1 Fig).

## Results

In this section, we evaluate the accuracy of msCentipede, using multiple DNase-seq and ATAC-seq data sets, on a set of transcription factors for which high quality ChIP-seq data and highly informative position-weight matrix (PWM) models are available. We also evaluate the gain in performance achieved when we use a more flexible model for background DNase I cleavage rate, with parameters for this model learned using DNase-seq data from naked DNA.

### msCentipede achieves improved accuracy

**Fig 2:**
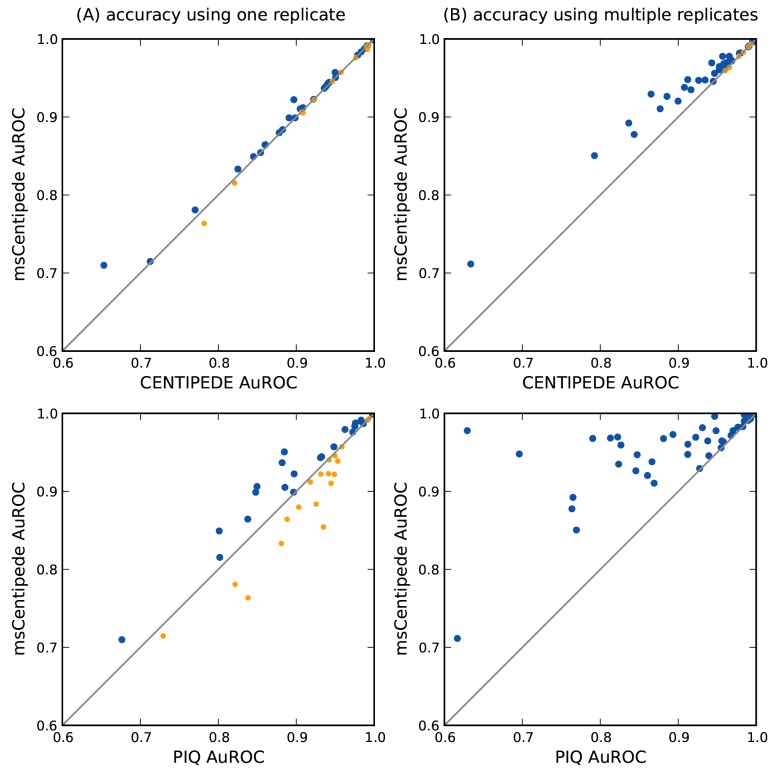
Accuracy of msCentipede, CENTIPEDE and PIQ across a range of transcription factors. Each point corresponds to a different factor and accuracy is measured by area under the ROC curve. Blue points correpond to factors where msCentipede achieves higher accuracy than CENTIPEDE (top panels) or PIQ (bottom panels), and orange points correspond to a worse performance by msCentipede. A: The algorithms are compared using data from a single replicate. B: The algorithms are compared using data from multiple library replicates.

msCentipede achieved AuROC comparable to or better than CENTIPEDE across a broad range of transcription factors when each algorithm was applied to chromatin accessibility measurements from a single DNase-seq assay as shown in Fig 2A (top). Compared with PIQ, we observed that msCentipede achieved substantially higher AuROC for some factors and lower AuROC for others, as shown in Fig 2A (bottom) (see S1 Table for more details). When multiple replicates are available, CENTIPEDE requires pooling the replicate datasets and PIQ uses the replicate datasets to jointly learn the background Gaussian process model; however, msCentipede treats replicates by modeling them as independent samples. By modeling the replicates appropriately and accounting for heterogeneity across genomic sites and replicates, msCentipede achieved substantial increase in AuROC compared to CENTIPEDE and PIQ for a broad range of transcription factors, as illustrated in Fig 2B. Similar improvements in accuracy for msCentipede compared to CENTIPEDE were observed when using ATAC-seq measurements as data (see S2 Fig).

For each transcription factor, the hyperparameter *τ* gives a measure of heterogeneity in read distribution across genomic sites and replicates, with lower values indicating greater heterogeneity. In S3 Fig, we observed that the values of the hyperparameters *τ*_*j*_ were rather small, suggesting that we were able to increase power by better modeling variation in the data. Furthermore, we observed a higher degree of overdispersion in read distribution at medium resolutions compared to the finest and coarsest resolutions across all transcription factors.

### Modeling DNase I cleavage patterns improves factor binding inference

In recent work, He et al. [16] and Sung et al. [25] demonstrated that strong DNA sequence preference for DNase I cleavage could pose a challenge to using the detailed shape of DNase cleavage profiles for inferring transcription factor binding. Specifically, He et al. [16] identified motif instances that lie within peaks in ChIP-seq measurements for a transcription factor in a given cell line. Using these instances, they showed that, in a region of ∼ 20 bp surrounding the motif, the mean DNase I cleavage profile estimated from naked DNA (unbound sites) matched the mean cleavage profile estimated using DNase-seq data from the same cell line (bound sites). Starting from similar observations, Sung et al. [25] clarified that although sequence-preference effects were evident for all transcription factors, some transcription factors—those with slower-binding kinetics—show an appreciable reduction in the cut profile around the bound motifs (a “footprint”), whereas others—those with faster-binding kinetics—show little or no footprint.

These observations raise two questions: first, whether the uniform background model (assumed by CENTIPEDE, and msCentipede) for the unbound sites might be better replaced by a non-uniform background model capable of capturing the sequence preference effects around the motifs; second, whether it might be better to entirely ignore the DNase I cleavage profile when attempting to distinguish between bound and unbound sites—and, rather, to focus only on the total intensity of DNase I hypersensitivity in the region. To test this, we compared the accuracy of three different models for transcription factor binding:

1. ‘no cleavage profile’ model that ignores the cleavage profile, and simply models the total DNase read counts using Poisson-gamma distributions at bound and unbound sites (described earlier).
2. msCentipede
3. msCentipede-flexbg, which allows for a non-uniform background model, with parameters estimated using DNase-seq measurements from naked DNA around the same set of PBS.

**Fig 3:**
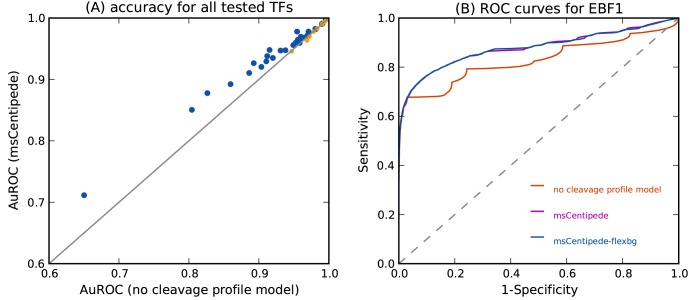
Modeling factor-specific DNase I cleavage profile and sequence bias in DNase cleavage increases prediction accuracy. A: Modeling the DNase I cleavage profile at bound sites increases the prediction accuracy of msCentipede across a broad range of transcription factors. Each point on the plot corresponds to a different transcription factor. B: We show the ROC curves for transcription factor EBF1 for three different models of increasing complexity. We observe a substantial increase in accuracy when incorporating a multi-scale model for the factor-specific cleavage profile; however, the increase in accuracy when modeling the background cleavage rate using naked DNA data is rather modest. This holds true for a broad range of factors as shown in S4 Fig.

Comparing first the msCentipede model with the no-cleavage model, we found the accuracy of msCentipede to be substantially greater for a broad range of transcription factors (Fig 3A). This result may appear to conflict with previous results [16, 25] showing that cleavage patterns within factor-bound motif instances are driven primarily by sequence preferences for DNase I cleavage, which suggests that use of the cleavage profile to identify binding sites could increase false positive findings. However, we note that i) sequence preference effects, while presumably occuring genome wide, are *shared* across binding sites only in the small region around the shared sequence motif (typically 10–20 bp), while most methods to detect factor binding, including ours, make use of cleavage patterns in much larger windows (typically 50–100 bp) around the motif instance, and ii) for some factors—those with slower binding kinetics—the footprint effect (i.e. the systematic overall decrease in DNase signal surrounding the motif) may be helpful in distinguishing bound and unbound sites, and the benefits of this could outweigh the unmodelled sequence preference effects.

We turn now to evaluate the effect of sequence bias in DNase I cleavage patterns on the inference of TF binding by comparing msCentipede with msCentipede-flexbg. Note that msCentipede-flexbg, by modeling the background cleavage profile using naked DNA assays, has the potential to eliminate false positives due to sequence-driven cleavage patterns highlighted earlier [16, 25]. And indeed, we found that, for most factors, the estimated mean background cleavage profile, captured by the parameters ${\bar{p}}_{jk}^{o}$, was non-uniform within the motif, reflecting precisely the sequence preferences for DNase I cleavage (S5 Fig). However, we also found that this improved background model resulted in only modest improvements in accuracy of identifying bound sites (S4 Fig). It is possible that accuracy could be further improved by explicitly modeling sequence-specific context effects in more detail than we have here, for example by relating cleavage rates at each location to the surrounding k-mers. However, our preliminary attempts to achieve this were unsuccessful (results not shown).

Using transcription factor EBF1 as an example, Fig 3B illustrates that all three models have very similar true positive rates up to a false positive rate of 3–4%. However, incorporating the DNase cleavage profile substantially increased the true positive rate for false positive rates larger than 4%. This suggests that while modeling the total DNase read counts alone was sufficient to accurately identify bound PBS with highest total DNase-seq signal, incorporation of the DNase cleavage profile was necessary to identify bound PBS with moderate total DNase-seq signal. These PBS may be indicative of low occupancy sites where the binding of the transcription factor is in a less stable equilibrium and the factor is likely bound to the DNA at these PBS in a smaller fraction of the cells assayed.

## Discussion

We developed msCentipede, a hierarchical multi-scale model to accurately identify binding of a transcription factor using sequencing reads from DNase-seq or ATAC-seq assays and the sequence content of putative binding sites for that factor in the genome. While previous approaches like CENTIPEDE have successfully used the characteristic profile of DNA hypersensitivity to DNase I around bound motif instances to identify factor binding sites, the multinomial model used in CENTIPEDE ignores spatial structure in the data and makes a strong assumption on the heterogeneity in read distribution across bound sites in the genome. Moreover, when multiple replicate measurements are available, CENTIPEDE ignores heterogeneity across replicates. The hierarchical multi-scale model explicitly allows for heterogeneity in the read distribution across bound sites and across replicate measurements (with different amounts at different scales), resulting in a substantial increase in accuracy across a broad range of transcription factors. Finally, we explored the effects of sequence bias in DNase I on inference by using a simple, flexible background model that can exploit the availability of DNase-seq data assayed in naked DNA. This flexible background model has the potential to account for heterogeneity in background DNase I cleavage rate specific to the sequence context of motif instances of the transcription factor.

A simple extension to CENTIPEDE that can account for heterogeneity across sites is to allow for site-specific parameters in the multinomial distribution and to model these site-specific parameters using a Dirichlet distribution. However, this multinomial-Dirichlet model is not sufficiently flexible to capture potential spatial structure in heterogeneity in DNase I cleavage, since it has only one additional parameter that captures variance. The proposed multi-scale model allows different amounts of variance across different scales, effectively capturing spatial structure in the heterogeneity. It is fairly straightforward to extend the proposed framework to model spatial structure in the mean cleavage pattern ($\bar{p}$) as usually modeled in multi-scale approaches [10, 11, 13]. However, we found that this extension was computationally expensive and gave very minor improvements in accuracy, presumably because there were so many motif instances that we could accurately estimate the mean pattern without spatial smoothing.

When considering a flexible model at unbound motif instances which allows for spatial structure and heterogeneity in background DNase I cleavage patterns, it is natural to estimate the parameters of this model using data from the relevant cell type. However, we observed that when all the parameters in the flexible model are estimated using data from chromatin, the model tended to estimate smaller values for the precision parameter, *τ*, at bound sites resulting in a large number of ‘true’ unbound sites being incorrectly identified as bound. Currently, we suggest using the flexible model (msCentipede-flexbg) only when DNase-seq (or ATAC-seq) data assayed in naked DNA is available. However, a framework that allows estimation of heterogeneity in background DNase I cleavage from data assayed in the relevant cell type may be be more accurate.

msCentipede-flexbg estimates spatial structure and heterogeneity in the background model using DNase-seq data from naked DNA at all motif instances; thus, the heterogeneity in background read distribution is primarily driven by variation in sequence context around motif instances. However, within a cell type, variation in background chromatin context at unbound sites (e.g., whether the motif instance is in the linker region or in DNA wrapped around a nucleosome, and which other transcription factors are bound at or close to the motif instance) is likely to be a larger source of heterogeneity in background read distribution than variation in sequence context. This intuition suggests that we should estimate the precision parameter at unbound sites *τ* ^*o*^ using DNase-seq data from chromatin, rather than using DNase-seq data from naked DNA. However, using this approach, we observed the background precision parameter *τ* ^*o*^ in msCentipede-flexbg was consistently underestimated when this parameter was estimated using data from chromatin, resulting in a high false positive rate. Extensions to these models that accurately capture the background heterogeneity in the data across genomic sites would be a useful avenue for future research.

## Supporting Information

S1 Fig Accuracy of msCentipede, CENTIPEDE and PIQ using a gold standard identified using MACS. Each point corresponds to a different factor and accuracy is measured by area under the ROC curve. Blue points correpond to factors where msCentipede achieves higher accuracy than CENTIPEDE (top panels) or PIQ (bottom panels), and orange points correspond to a worse performance by msCentipede.
(EPS)

S2 Fig Accuracy of msCentipede and CENTIPEDE using ATAC-seq data and a gold standard identified using GEM. Blue points correpond to factors where msCentipede achieves higher accuracy than CENTIPEDE and orange points correspond to a worse performance by msCentipede.
(EPS)

S3 Fig Heterogeneity across different scales. A: A plot of the precision parameter *τ* as a function of the scale in the multi-scale model. Each gray line corresponds to a different transcription factor and the solid blue line shows the median trend across all factors. B: A plot of the relative change in AuROC as a function of the lower bound on the precision parameter *τ* for all scales. Each line corresponds to a transcription factor, and red lines correspond to factors where most lower bounds lead to a decrease in accuracy. Although the AuROC is fairly robust to the maximum allowed dispersion (minimum allowed precision), most factors show a modest decrease in accuracy for higher values of the lower bound.
(EPS)

S4 Fig Evaluating the effect of DNase I sequence bias. A: Comparing the accuracy of msCentipede and msCentipede-flexbg. Blue points correspond to factors where msCentipede-flexbg shows improved performance and orange points correspond to a worse performance by msCentipede-flexbg. The increase in accuracy for msCentipede-flexbg is relatively modest across a large number of transcription factors. B: Comparing the accuracy of msCentipede-flexbg with fixed zero-variance in the background model, and msCentipede-flexbg. Most of the improvement of msCentipede-flexbg over msCentipede arises from modeling variance in the background DNase I cleavage patterns.
(EPS)

S5 FigNormalized DNase I cleavage profiles in chromatin and naked DNA, for a subset of transcription factors.The cleavage profiles for chromatin and naked DNA were computed from the maximum likelihood estimates of the parameters ${\bar{p}}_{jk}$ and ${\bar{p}}_{jk}^{o}$, respectively. For the sake of clarity, only the plus strand cleavage profile is shown. The dotted orange lines indicate the boundaries of the core motif.(EPS)

S6 Fig Accuracy of msCentipede and CENTIPEDE at different sequencing depths. msCentipede achieves better (or similar) accuracies as CENTIPEDE for majority of the TFs in each of the replicates, indicating that the results hold across almost a 5-fold difference in coverage. Indeed, as the sequencing depth approaches 100 million reads, we observe the accuracy of CENTIPEDE and msCentipede to be highly concordant, while msCentipede achieves higher accuracies at sequencing depths closer to 10 million reads.
(EPS)

S7 Fig Accuracy of msCentipede, CENTIPEDE and PIQ on pooled replicates. Accuracy of msCentipede is similar to that of CENTIPEDE, and substantially better than that of PIQ, when applied to pooled replicate data. Note that msCentipede applied to pooled data achieves worse accuracy than when the replicates are treated as independent samples (despite the total sequencing depth being the same), since the variance across replicates is not properly accounted for when pooling replicate data sets.
(EPS)

S8 Fig True positive rate of msCentipede, CENTIPEDE and PIQ at 5% false positive rate. msCentipede achieves better (or similar) true positive rates compared to CENTIPEDE, and substantially higher true positive rates than PIQ. Multiple DNase replicates were used in this analysis, and msCentipede and PIQ were run in their multi-replicate modes.
(EPS)

S1 Methods Detailed description of the model. A detailed description of the model for msCentipede, along with the estimation and inference framework.
(PDF)

S1 Table Accuracy when using one DNase-seq replicate. A list of the transcription factors, their PWM models and the AUC score achieved by the different algorithms listed in the main text, using only one DNase-seq data set. Factors for which PIQ achieves a higher accuracy than msCentipede are highlighted in red.
(PDF)

S2 Table Accuracy when using multiple DNase-seq replicates. A list of the transcription factors, their PWM models and the AUC score achieved by the different algorithms listed in the main text, using both replicate DNase-seq data sets. Factors for which PIQ achieves a higher accuracy than msCentipede are highlighted in red.
(PDF)
