# Supplementary material for: msCentipede: Modeling Heterogeneity across Genomic Sites and Replicates Improves Accuracy in the Inference of Transcription Factor Binding
Source: PLoS One. 2015 Sep 25;10(9):e0138030. doi: 10.1371/journal.pone.0138030 (PMC4583425; doi:10.1371/journal.pone.0138030)
Supplement: S1 Methods — A detailed description of the model for msCentipede, along with the estimation and inference framework. (PDF) [file pone.0138030.s009.pdf]

Supplementary Methods

1 Quantifying heterogeneity in DNase I cleavage patterns

We made use of the connection between the multinomial distribution and binomial distribution to explore the amount of heterogeneity in DNase I cleavage patterns across genomic sites. Specifically, if the read counts per base pair are multinomially distributed conditional on the total read count in a genomic window, then the number of reads mapping to the left half of the window conditional on total read count should be binomially distributed. Based on this, we compared the true distribution of the proportion of reads mapping to the left half of a genomic window to a distribution of proportions computed by sampling read counts from a binomial distribution.

To illustrate, we considered the transcription factor SP1, and focused on the 1000 motif instances with the highest ChIP-seq signal and with at least 10 DNase-seq reads mapped to a 100 bp window around the motif instance. For each window, we computed the 'true' proportion of reads mapping to the left half of the window. Next, assuming that the count on the left half conditional on the total count is binomially distributed, we computed the maximum likelihood estimate of the binomial parameter $\hat{p}$ using data from these windows. Finally, for each window, we sampled a read count for the left half conditional on the total count for that window from a binomial distribution whose parameter was set to be the maximum likelihood estimate $\hat{p}$, and computed a 'simulated' proportion of reads mapping to the left half of the window. Histograms of the 'true' proportions and 'simulated' proportions are shown in Figure 1 in the main text.

2 msCentipede model for one replicate

Consider a genomic window of length L around each of N putative binding sites. We define $X^n = (X_l^n)_{l=1}^L$ for $n = 1, \ldots, N$, where X_l^n is read count at l^{th} base pair in the window around the nth site. Let Z^n denote a binary indicator for whether the nth site is bound ($Z^n = 1$). A mixture model at the nth site can be written as

$$\mathsf{P}(X^n) = \mathsf{P}(X^n|Z^n = 1)\mathsf{P}(Z^n = 1) + \mathsf{P}(X^n|Z^n = 0)\mathsf{P}(Z^n = 0), \tag{1}$$

where the prior probability $\mathsf{P}(Z^n = 1) = \zeta_n$ can be modeled as a logistic function of genomic information (e.g. position weight matrix score and sequence conservation score of the motif instance) as in CENTIPEDE. Specifically, if $S_n = \{S_{n1}, \ldots, S_{nF}\}$ is a vector representing various genomic features for the nth site, then $\zeta_n = \frac{1}{1+e^{-\sum_f \gamma_f S_{nf}}}$, where γ_f captures how much the fth feature informs the prior probability that a site is bound by a transcription factor.

At the zeroth scale, we model the total number of reads in the entire region $Y_{01}^n := \sum_l X_l^n$ as follows:

$$Y_{01}^n \sim \text{Poisson}(\lambda^n). \tag{2}$$

At the first scale, conditional on Y_{01}^n, we model the number of reads in the first half of the region $Y_{11}^n := \sum_{l \le L/2} X_l^n$ using a binomial distribution, $Y_{11}^n \mid Y_{01}^n \sim \text{binomial}(Y_{01}^n, p_{01}^n)$. Then, the number of reads in the second half of the region is determined to be $Y_{12}^n := Y_{01}^n - Y_{11}^n$. At the second scale, conditional on Y_{11}^n, we model the read count in the first quarter of the region Y_{21}^n using a binomial distribution, $Y_{21}^n \mid Y_{11}^n \sim \text{binomial}(Y_{11}^n, p_{11}^n)$, leading to the read count in the second quarter of the region $Y_{22}^n := Y_{11}^n - Y_{21}^n$.

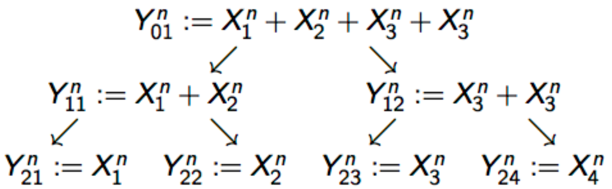

Figure S1: Multi-scale models for inhomogeneous Poisson processes. Suppose we have observations $X^n = (X_l^n)_{l=1}^4$ on four bases. Multi-scale models assume that $Y_{01}^n \sim \text{Poisson}(\mu^n)$, $Y_{11}^n \mid Y_{01}^n \sim \text{binomial}(Y_{01}^n, p_{01}^n)$ (then, $Y_{12}^n = Y_{01}^n - Y_{11}^n$), $Y_{21}^n \mid Y_{11}^n \sim \text{binomial}(Y_{11}^n, p_{11}^n)$ (then, $Y_{22}^n = Y_{11}^n - Y_{21}^n$), and $Y_{23}^n \mid Y_{12}^n \sim \text{binomial}(Y_{12}^n, p_{12}^n)$ (then, $Y_{24}^n = Y_{12}^n - Y_{23}^n$). It follows from elementary properties of the Poisson distribution (see [2] and [3] for details) that it is equivalent to $Y_{2l}^n = X_l^n \sim \text{Poisson}(\mu_l^n)$ for $l = 1, 2, 3, 4$, where $\mu_1^n = \lambda^n p_{01}^n p_{11}^n$, $\mu_2^n = \lambda^n p_{01}^n (1 - p_{11}^n)$, $\mu_3^n = \lambda^n (1 - p_{01}^n) p_{12}^n$, and $\mu_4^n = \lambda^n (1 - p_{01}^n)(1 - p_{12}^n)$.

Conditional on Y_{12}^n, the read count in the third quarter of the region Y_{23}^n is modeled using a binomial distribution, $Y_{23}^n \mid Y_{12}^n \sim \text{binomial}(Y_{12}^n, p_{12}^n)$, leading to the read count in the fourth quarter of the region $Y_{24}^n := Y_{12}^n - Y_{23}^n$. This process continues through the scales: at the Jth scale (finest-resolution), there are $L = 2^J$ parts of the region. The read counts in the lth part of the region Y_{Jl}^n, which is equivalent to X_l^n, is modeled as follows:

$$Y_{J,2k-1}^n \mid Y_{J-1,k}^n \sim \text{binomial}(Y_{J-1,k}^n, p_{J-1,k}^n), \tag{3}$$
$$Y_{J,2k}^n = Y_{J-1,k}^n - Y_{J,2k-1}^n, \tag{4}$$

for $k = 1, \ldots, 2^{J-1}$ (see Figure S1 for a brief illustration of the model).

When $Z^n = 1$, heterogeneity across genomic sites is modeled as follows:

$$\lambda^n | Z^n = 1 \sim \text{gamma}\left(\alpha, \alpha/\bar{\lambda}_0\right), \tag{5}$$
$$p_{jk}^n | Z^n = 1 \sim \text{beta}(\bar{p}_{jk}\tau_j, (1 - \bar{p}_{jk})\tau_j), \tag{6}$$

where α and $\bar{\lambda}_0$ capture the mean ($\bar{\lambda}_0$) and variance ($\frac{\bar{\lambda}_0^2}{\alpha}$) in overall DNase I hypersensitivity of TF bound genomic locations, and p_{jk} and τ_j capture the mean and variance in the DNase I cleavage profiles across TF bound genomic locations. When $Z^n = 0$, we model heterogeneity in total DNase I hypersensitivity as follows:

$$\lambda^n | Z^n = 0 \sim \text{gamma}\left(\alpha^o, \alpha^o/\bar{\lambda}_0^o\right), \tag{7}$$
$$p_{jk}^n | Z^n = 0 \sim \delta_{0.5}. \tag{8}$$

3 msCentipede model for multiple replicates

Suppose we have S replicate DNase-seq measurements for a particular cell type. Given a genomic window of length L around each of N putative binding sites, we define $X^{n,s} = (X_l^{n,s})_{l=1}^L$ for $n = 1, \ldots, N$ and $s = 1, \ldots, S$, where $X_l^{n,s}$ is read count at l^{th} base pair in the window around the nth site for the sth replicate.

In the mixture model specified earlier, conditional on $Z^n = 1$, we can model the total number of reads in the entire region $Y_{01}^{n,s} := \sum_l X_l^{n,s}$ as follows:

$$Y_{01}^{n,s} \sim \text{Poisson}(\lambda^{n,s}), \tag{9}$$
$$\lambda^{n,s} | Z^n = 1 \sim \text{gamma}\left(\alpha^s, \alpha^s / \bar{\lambda}_0^s\right), \tag{10}$$

where replicate-specific parameters, α^s and $\bar{\lambda}_0^s$, capture replicate-specific mean ($\bar{\lambda}_0^s$) and variance ($\frac{\bar{\lambda}_0^{s2}}{\alpha^s}$). At the remaining scales, $Y_{jk}^{n,s}$, $k = 1, \dots, 2^{j-1}$ and $j = 1, \dots, J$, conditional on $Y_{01}^{n,s}$ and $Z^n = 1$ is modified as follows:

$$Y_{j,2k-1}^{n,s} \mid Y_{j-1,k}^{n,s} \sim \text{binomial}(Y_{j-1,k}^{n,s}, p_{jk}^{n,s}), \tag{11}$$
$$p_{jk}^{n,s} | Z^n = 1 \sim \text{beta}(\bar{p}_{jk}\tau_j, (1 - \bar{p}_{jk})\tau_j). \tag{12}$$

Ideally, it is desirable to model the variation across genomic sites and the variation across replicates separately. However, in practice, we usually have only two or three replicate DNase-seq measurements in a given cell type, making it difficult to accurately quantify the variation across replicates. Instead, we assume that variation across replicates and variation across genomic sites are the same. The background model $\mathsf{P}(X^n | Z^n = 0)$ can be constructed in a similar way.

4 Maximum likelihood estimation and inference

Inference for the above model requires computing the posterior distribution $\mathsf{P}(Z|X)$, evaluated at the maximum likelihood estimate of the model parameters Θ^*, where $\Theta = \{\bar{p}_{jk}, \tau_j, \alpha^s, \bar{\lambda}_0^s, \alpha_o^s, \bar{\lambda}_{0o}^s\}$. This problem is equivalent to finding the optimal distribution in a parameteric family of distributions $q(Z)$ that has the smallest Kullback-Leibler (KL) divergence to the posterior distribution of interest.

$$q^*(Z) = \arg\min_{q(Z)} \sum_Z q(Z) \log \frac{q(Z)}{\mathsf{P}(Z|X)} \tag{13}$$
$$= \log \mathsf{P}(X; \Theta) - \arg\max_{q(Z)} \left[\sum_Z q(Z) \log \frac{\mathsf{P}(X|Z, \Theta)\mathsf{P}(Z)}{q(Z)} \right] \tag{14}$$
$$= \log \mathsf{P}(X; \Theta) - \arg\max_{q(Z)} \mathcal{F}[q(Z); \Theta]. \tag{15}$$

The first term in 15 is the log likelihood of the data for some fixed value for the model parameters Θ. Thus, minimizing the KL divergence is equivalent to maximizing the function $\mathcal{F}[q(Z); \Theta]$, keeping the model parameters fixed. Note that, when the true posterior distribution $\mathsf{P}(Z|X)$ lies in the specified parametric family, the maximum value $\mathcal{F}[q^*(Z); \Theta]$ is equal to the log likelihood of the data $\log \mathsf{P}(X; \Theta)$ (i.e., the minimum KL divergence is zero). The maximum likelihood estimate of the model parameters Θ can then be obtained by maximizing the function $\mathcal{F}[q^*(Z); \Theta]$. Maximizing $\mathcal{F}[q(Z); \Theta]$ with respect to $q(Z)$ and Θ, iteratively till convergence, gives us the maximum likelihood estimate of the model parameters and posterior probability of transcription factor binding.

Conditional on the model parameters, the data at all putative binding site in all replicates are independent. Thus, the true posterior $\mathsf{P}(Z|X)$ will factorize as $\mathsf{P}(Z|X) = \prod_n \mathsf{P}(Z^n|X^n)$, motivating the following

choice for $q(Z)$.

$$q(Z) = \prod_n \text{Bernoulli}(\tilde{\zeta}_n). \tag{16}$$

The function to be maximized can now be written as follows:

$$\mathcal{F}[q(Z);\Theta] = \sum_n \sum_{Z^n} q(Z^n) \log \mathsf{P}(X^n|Z^n,\Theta) + \sum_n \sum_{Z^n} q(Z^n) \log \frac{\mathsf{P}(Z^n)}{q(Z^n)} \tag{17}$$

$$= \sum_n \tilde{\zeta}_n \log \mathsf{P}(X^n|Z^n = 1,\Theta) + (1-\tilde{\zeta}_n) \log \mathsf{P}(X^n|Z^n = 0,\Theta) \tag{18}$$

$$+ \tilde{\zeta}_n \log \frac{\zeta_n}{\tilde{\zeta}_n} + (1-\tilde{\zeta}_n) \log \frac{1-\zeta_n}{1-\tilde{\zeta}_n} \tag{19}$$

Maximizing $\mathcal{F}$ with respect to $\tilde{\zeta}_n$ for fixed Θ gives

$$\log \frac{\tilde{\zeta}_n^*}{1-\tilde{\zeta}_n^*} \equiv \log \frac{\mathsf{P}(Z^n = 1|X^n)}{1-\mathsf{P}(Z^n = 1|X^n)} = \log \frac{\zeta_n}{1-\zeta_n} + \log \frac{\mathsf{P}(X^n|Z^n = 1,\Theta)}{\mathsf{P}(X^n|Z^n = 0,\Theta)} \tag{20}$$

This equation gives us the posterior probability that a site is bound, and is equivalent to the E-step in the EM algorithm. Using the model specified in the previous section, we have

$$\log \mathsf{P}(X^n|Z^n = 1,\Theta) = \sum_s \log \mathsf{P}(Y_{01}^{n,s}|\alpha^s,\bar{\lambda}_0^s) + \sum_s \sum_{j=1}^{J} \sum_{k=1}^{2^{(j-1)}} \log \mathsf{P}(Y_{j,2k-1}^{n,s}|Y_{j-1,k}^{n,s},\bar{p}_{jk},\tau_j), \tag{21}$$

where

$$\mathsf{P}(Y_{01}^{n,s}|\alpha^s,\bar{\lambda}_0^s) = \text{negative binomial}\left(\alpha^s, \frac{\bar{\lambda}_0^s}{\alpha^s+\bar{\lambda}_0^s}\right), \tag{22}$$

$$\mathsf{P}(Y_{j,2k-1}^{n,s}|Y_{j-1,k}^{n,s},\bar{p}_{jk},\tau_j) = \text{beta binomial}(Y_{j-1,k}^{n,s};\bar{p}_{jk}\tau_j,(1-\bar{p}_{jk})\tau_j). \tag{23}$$

A similar likelihood function for the background model $\log \mathsf{P}(X^n|Z^n = 0,\Theta)$ can be written.

Keeping $q(Z)$ fixed, the function $\mathcal{F}$ can be maximized with respect to the model parameters Θ, within appropriate constraints, using standard optimization solvers. By iterating between computing the posterior probability $q(Z)$ (at fixed Θ) and optimizing $\mathcal{F}$ (keeping $q(Z)$ fixed), until convergence, we get the maximum likelihood estimates of the parameters Θ. In this sense, the iterative variational optimization scheme described above is equivalent to the EM algorithm for computing the maximum likelihood estimates of the parameters.

In the current version of the software, we optimize the model parameters Θ using cvxopt [1], a standard primal-dual interior-point optimization solver. In addition, by treating the simple iterative algorithm above as a fixed-point solver, we can accelerate its convergence [4]. While this accelerated version reaches the optimum using fewer evaluations of the gradient and hessian of $\mathcal{F}$, this gain is at the expense of the monotonicity guaranteed by the simple iterative algorithm.

References

[1] M. S. Andersen, J. Dahl, and L. Vandenberghe. CVXOPT: A Python package for convex optimization, version 1.1.7. 2014.

[2] Eric D. Kolaczyk. Bayesian multiscale models for Poisson processes. *Journal of the American Statistical Association*, 94(447):920–933, September 1999.

[3] K.E. Timmermann and R.D. Nowak. Multiscale modeling and estimation of Poisson processes with application to photon-limited imaging. *IEEE Transactions on Information Theory*, 45(3):846–862, April 1999.

[4] R Varadhan and C Roland. Simple and globally convergent methods for accelerating the convergence of any em algorithm. *Scandinavian Journal of Statistics*, 35(2):335–353, 2008.
