# Supplementary material for: msCentipede: Modeling Heterogeneity across Genomic Sites and Replicates Improves Accuracy in the Inference of Transcription Factor Binding
Source: PLoS One. 2015 Sep 25;10(9):e0138030. doi: 10.1371/journal.pone.0138030 (PMC4583425; doi:10.1371/journal.pone.0138030)
Supplement: S1 Table — A list of the transcription factors, their PWM models and the AUC score achieved by the different algorithms listed in the main text, using only one DNase-seq data set. Factors for which PIQ achieves a higher accuracy than msCentipede are highlighted in red. (PDF) [file pone.0138030.s010.pdf]

Table S1

A list of the transcription factors, their PWM models and the AUC score achieved by the different algorithms listed in the main text, using only one DNase-seq data set. Factors for which PIQ achieves a higher accuracy than msCentipede are highlighted in red.

factor	PWM model	CENTIPEDE	msCentipede	PIQ
BATF	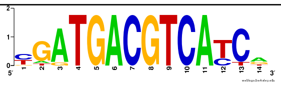	0.897	0.922	0.931
BHLHE40	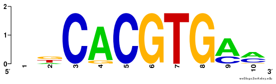	0.899	0.899	0.848
CEPBP	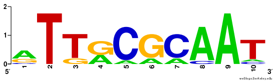	0.958	0.957	0.959
CTCF	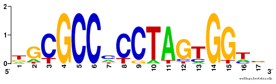	0.891	0.899	0.897
E2F4	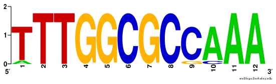	0.993	0.993	0.993
EBF	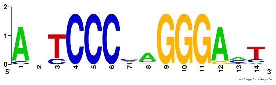	0.782	0.763	0.838
EGR1	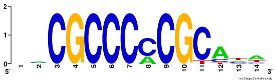	0.938	0.939	0.953
ELF1	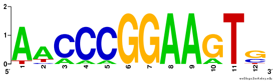	0.942	0.944	0.933
ELK1	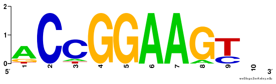	0.983	0.983	0.975
ELK1	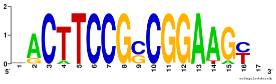	0.976	0.976	0.972
ERRA	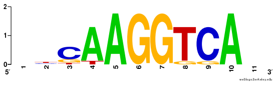	0.909	0.905	0.885
ERRA	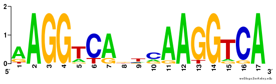	0.946	0.946	0.950
ETS1	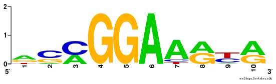	0.991	0.991	0.992
ETS1	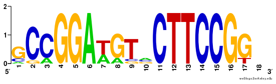	0.999	0.999	0.998
GABP	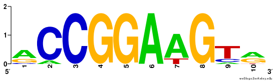	0.979	0.979	0.963
IRF3	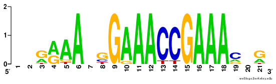	0.922	0.923	0.941

IRF4	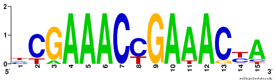	0.950	0.957	0.948
MAFK	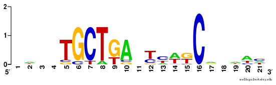	0.653	0.710	0.676
MAX	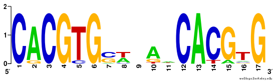	0.936	0.937	0.882
MEF2A	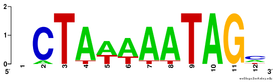	0.821	0.815	0.802
NFATC1	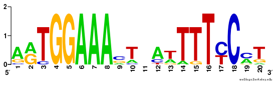	0.854	0.854	0.935
NFATC1	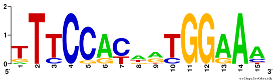	0.878	0.880	0.903
NFE2	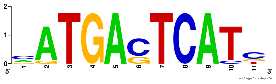	0.950	0.951	0.885
NFKB	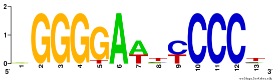	0.907	0.906	0.850
NRF1	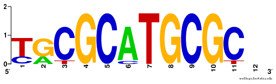	0.990	0.990	0.983
PAX5	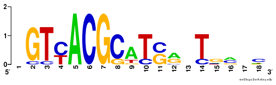	0.845	0.849	0.801
POU2F2	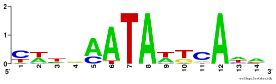	0.909	0.912	0.918
PU1	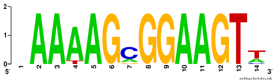	0.713	0.715	0.729
RFX5	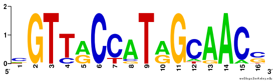	0.939	0.940	0.942
RUNX3	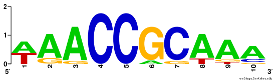	0.825	0.833	0.881
RUNX3	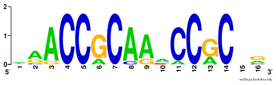	0.860	0.864	0.838
RXRA	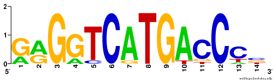	0.923	0.922	0.949
SP1	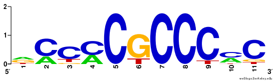	0.991	0.987	0.986
SREBP1	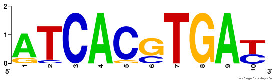	0.988	0.988	0.976
SREBP2	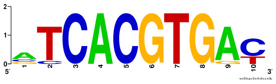	0.991	0.991	0.983

SRF	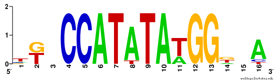	0.770	0.781	0.822
TCF3	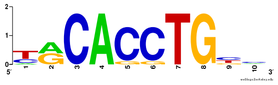	0.923	0.922	0.897
TR4	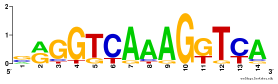	0.905	0.910	0.945
USF1	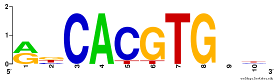	0.860	0.864	0.888
YY1	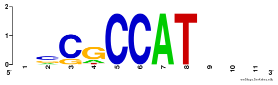	0.882	0.884	0.926
ZNF143	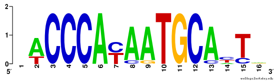	0.941	0.943	0.931
