# Supplementary material for: msCentipede: Modeling Heterogeneity across Genomic Sites and Replicates Improves Accuracy in the Inference of Transcription Factor Binding
Source: PLoS One. 2015 Sep 25;10(9):e0138030. doi: 10.1371/journal.pone.0138030 (PMC4583425; doi:10.1371/journal.pone.0138030)
Supplement: S2 Table — A list of the transcription factors, their PWM models and the AUC score achieved by the different algorithms listed in the main text, using both replicate DNase-seq data sets. Factors for which PIQ achieves a higher accuracy than msCentipede are highlighted in red. (PDF) [file pone.0138030.s011.pdf]

Table S2

A list of the transcription factors, their PWM models and the AUC score achieved by the different algorithms listed in the main text, using both replicate DNase-seq data sets. Factors for which PIQ achieves a higher accuracy than msCentipede are highlighted in red.

factor	PWM model	no cleavage profile	CENTIPEDE	msCentipede	msCentipede-flexbg	PIQ
BATF	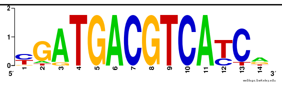	0.960	0.943	0.969	0.969	0.922
BHLHE40	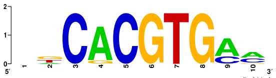	0.938	0.934	0.947	0.947	0.912
CEBPB	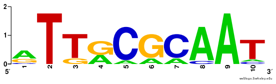	0.981	0.978	0.982	0.984	0.931
CTCF	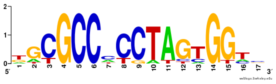	0.911	0.865	0.929	0.932	0.927
E2F4	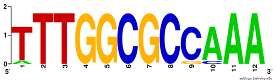	0.993	0.993	0.993	0.993	0.993
EBF	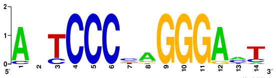	0.826	0.843	0.878	0.878	0.764
EGR1	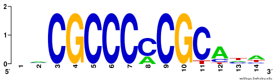	0.949	0.947	0.956	0.956	0.955
ELF1	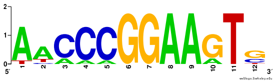	0.956	0.953	0.965	0.965	0.938
ELK1	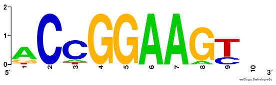	0.984	0.984	0.983	0.983	0.983
ELK1	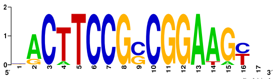	0.978	0.978	0.978	0.978	0.970
ERRA	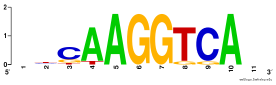	0.958	0.961	0.959	0.960	0.826
ERRA	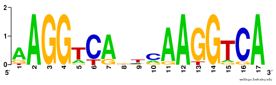	0.969	0.969	0.972	0.973	0.968
ETS1	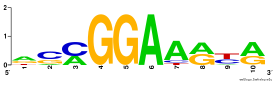	0.996	0.996	0.997	0.997	0.993
ETS1	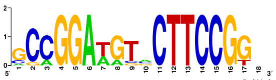	0.999	0.999	0.999	0.999	0.998
GABP	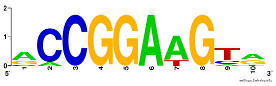	0.981	0.980	0.982	0.982	0.976
IRF3	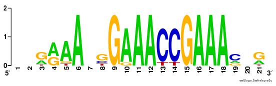	0.957	0.957	0.965	0.965	0.956

IRF4	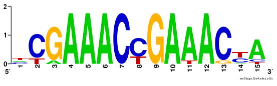	0.998	0.998	0.998	0.998	0.985
MAFK	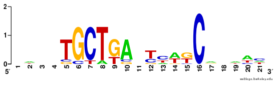	0.650	0.634	0.711	0.701	0.617
MAX	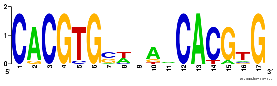	0.971	0.968	0.973	0.974	0.893
MEF2A	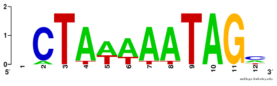	0.915	0.912	0.948	0.948	0.696
NFATC1	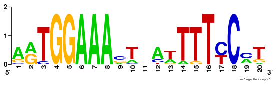	0.962	0.958	0.968	0.974	0.813
NFATC1	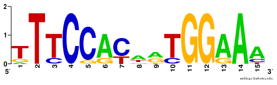	0.972	0.962	0.970	0.981	0.822
NFE2	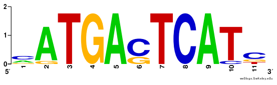	0.969	0.966	0.963	0.963	0.958
NFKB	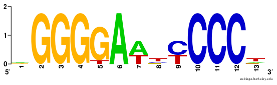	0.953	0.952	0.960	0.962	0.912
NRF1	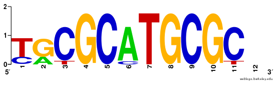	0.991	0.991	0.991	0.991	0.990
PAX5	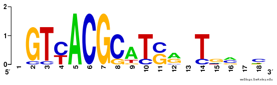	0.893	0.886	0.926	0.930	0.846
POU2F2	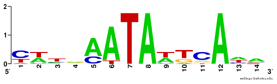	0.971	0.965	0.978	0.981	0.629
RFX5	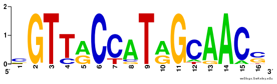	0.947	0.945	0.946	0.948	0.939
RUNX3	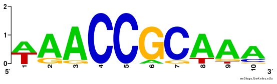	0.920	0.916	0.935	0.935	0.824
RUNX3	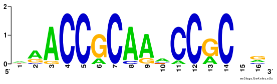	0.931	0.926	0.947	0.951	0.847
RXRA	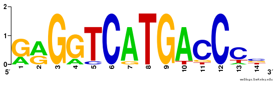	0.955	0.957	0.978	0.977	0.948
SP1	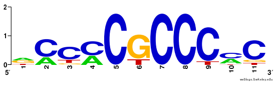	0.996	0.996	0.996	0.996	0.947
SPI1	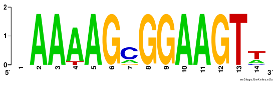	0.804	0.792	0.850	0.855	0.769
SREBP1	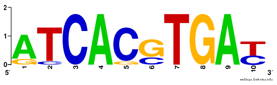	0.990	0.990	0.990	0.990	0.985
SREBP2	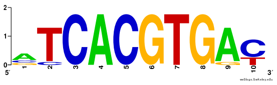	0.990	0.990	0.990	0.990	0.986

SRF	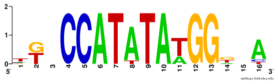	0.860	0.836	0.892	0.907	0.765
TCF3	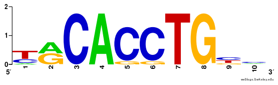	0.964	0.962	0.968	0.971	0.790
TR4	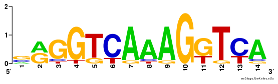	0.904	0.900	0.920	0.924	0.861
USF1	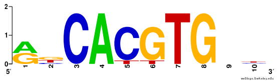	0.886	0.877	0.911	0.910	0.869
YY1	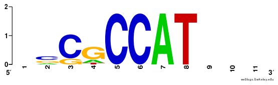	0.912	0.908	0.938	0.938	0.866
ZNF143	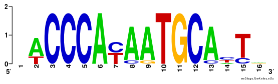	0.961	0.960	0.968	0.968	0.881
